# Factors associated with a diagnosis of sarcoidosis among US veterans of Iraq and Afghanistan

**DOI:** 10.1038/s41598-022-24853-8

**Published:** 2022-12-21

**Authors:** Nisha Jani, Israel C. Christie, Tianshi David Wu, Daniel E. Guzman, Jaehwan Han, Bryan Broderick, Michael J. Falvo, Anays Sotolongo, Omowunmi Y. Osinubi, Drew A. Helmer

**Affiliations:** 1 Airborne Hazards and Burn Pits Center of Excellence, War Related Illness and Injury Study Center, VA New Jersey Health Care System, East Orange, NJ USA; 2 New Jersey Medical School, Rutgers-The State University, Newark, NJ USA; 3 Rutgers University, School of Public Health, Newark, NJ USA; 4 Center for Innovations in Quality, Effectiveness and Safety (IQuESt), Michael E. DeBakey VA Medical Center, 2002 Holcombe Blvd (152), Houston, TX 77030 USA; 5 Department of Medicine, Baylor College of Medicine, Houston, TX USA; 6 Columbia University Irving Medical Center, New York, NY USA; 7 Johns Hopkins University School of Medicine, Baltimore, MD USA

**Keywords:** Risk factors, Respiratory signs and symptoms, Occupational health

## Abstract

This study evaluated risk factors of sarcoidosis among Airborne Hazards and Open Burn Pit Registry (AHOBPR) participants using a retrospective age and sex-matched case–control design of AHOBPR participants deployed to Afghanistan or Southwest Asia with and without sarcoidosis diagnosed in the Veterans Health Administration (VHA). Logistic regression models tested for associations between sarcoidosis and self-reported cumulative deployment-related exposures. 661 Veterans (0.37%) were diagnosed with sarcoidosis in VHA. Logistic regression demonstrated lower odds of sarcoidosis in Hispanic participants (OR 0.08, CI 0.04–0.15) and those who served in the Navy (OR 0.40, CI 0.21–0.72). African American veterans (OR 2.27, CI 1.66–3.11) and former smokers (OR 1.87, CI 1.33–2.62) were at elevated risk. Of the exposure variables, convoy activities had the highest odds of being associated with sarcoidosis and was marginally statistically significant (OR 1.16, CI 1.00–1.35). Sarcoidosis was an uncommon diagnosis among AHOBPR participants and was associated with only one of eight assessed cumulative deployment-related exposures.

## Introduction

Sarcoidosis is an uncommon condition with a prevalence of 60 per 100,000 in the United States^1^. Patients often present with respiratory symptoms such as cough, dyspnea, and chest pain accompanied by fatigue, malaise, fever, and weight loss^2^. Definitive diagnosis requires chest radiographic evidence accompanied by noncaseating granulomas on biopsy, with all other causes of granulomas ruled out^3^. Sarcoidosis affects people of all racial and ethnic groups and occurs at all ages, although it usually develops before the age of 50 years, with the incidence peaking at 20–39 years. It is more common among African Americans with an adjusted annual incidence roughly three times that among white Americans (35.5 cases per 100,000, as compared with 10.9 per 100,000)^4^. Women are also more likely to have sarcoidosis across racial and ethnic groups^5^. Low income and other financial barriers to care are significantly associated with sarcoidosis severity at presentation even after adjusting for demographic characteristics of race, sex, and age^6^.

Several factors have been associated with sarcoidosis including environmental and occupational exposures, infection, genetics, and epigenetics. It appears likely that the interplay of these factors triggers and sustains sarcoidosis. For example, numerous studies have consistently linked human leukocyte antigen genes to sarcoidosis and genome wide association studies have identified a few disease-risk alleles^7,8^. More recent exploration has supported a role for the epigenome in sarcoidosis, modulating the transcription of genes, perhaps in response to environmental factors, and initiating or perpetuating disease^9^. A 2016 meta-analysis involving more than 6000 patients from various countries worldwide suggests a significant association between sarcoidosis and some infectious agents, based on the marked difference in the percentage of microbial DNA-positive samples in sarcoidosis patients versus controls, especially mycobacteria (odds ratio (OR) 6.8) and *P*. *acnes* (OR 18.80)^10^. Both organic and inorganic materials have been found to be associated with sarcoidosis, especially in occupational cohorts^11^. Culprit materials include silica and silicates, metals, dust from the World Trade Center destruction, and wood combustion products identified through epidemiologic investigation and case reports^12–15^.

Because of unusual or even unique military exposures, sarcoidosis may be more common among US military service members and veterans, and risk may vary by military branch, occupation, and cohort of service. For example, elevated rates of sarcoidosis rates were detected in the 1960s and 1970s (the Vietnam era) among Navy personnel along with a decline in the African American/White ratio of these rates from about 17:1 to 6:1. The same analysis detected a lower risk of sarcoidosis among men who worked only on “clean ships”. These findings suggest that environmental exposures may cause (or increase one’s susceptibility to) sarcoidosis-like disease in naval settings^16^. A separate race-stratified analysis of 27 years of Navy personnel data found higher frequencies of sarcoidosis among aviation structural mechanics and food service personnel^17^. The authors suggest that a dust or moisture-related lung disease may have been erroneously classified as sarcoidosis, or, alternatively, that sarcoidosis may be associated with an unspecified occupational exposure^17^. Another analysis indicated that the incidence of sarcoidosis declined among naval enlisted men during 1965–1993, particularly among African-American men, and that the risk for sarcoidosis was statistically associated with the assignment of naval enlisted men to aircraft carriers^18^. Overall, the literature supports the possibility of a higher risk of sarcoidosis among military service members with a past elevated risk among certain naval occupational specialties but does not pinpoint a specific exposure trigger.

The US Department of Veterans Affairs (VA) and Department of Defense (DoD) Airborne Hazards and Open Burn Pit Registry (AHOBPR) is a voluntary online questionnaire available to veterans and active military service members who deployed to Southwest Asia or Djibouti after 1990 or Afghanistan after 2001. The questionnaire addresses several items related to exposures of potential relevance to sarcoidosis, including burn pit smoke and sandstorm exposure, construction activities, and refueling and engine maintenance activities. We utilize AHOBPR data to identify U.S. veteran participants deployed after 2001 with a sarcoidosis diagnosis in the Veterans Health Administration (VHA) and age matched controls to identify military exposure and other factors associated with sarcoidosis in this subpopulation of veterans.

## Methods

### Study data, period, and population

This analysis draws from data for 212,427 individuals who were deployed to Southwest Asia, Afghanistan, or Djibouti after October 7, 2001, and completed the online AHOBPR questionnaire as of August 15, 2020.

### Ethics declaration

The project was reviewed by the Institutional Review Board and the Research and Development Committee of the VA-New Jersey Health Care System. These boards granted a waiver of informed consent for this secondary analysis of data collected for clinical purposes. All analyses were conducted in accordance with relevant guidelines and regulations.

### Study variables

#### Sarcoidosis

The study population is AHOBPR participants who completed the online questionnaire between April 1, 2014, and August 15, 2020, who had records in the Veteran Health Administration. A case of sarcoidosis was defined as an AHOBPR participant with one or more International Classification of Diseases (ICD) version 9 or 10 codes (ICD9: 135; ICD10: D86.0–D86.9) for sarcoidosis in VHA records between 2011–2020. Controls were defined as AHOBPR participants with one or more VHA encounters between 2011–2020 and no documented sarcoidosis ICD codes. Optimal matching on age, stratified by gender, was performed at a 1:4 ratio to obtain the controls^19^.

#### Deployment-related exposure variables

Respondents listed all deployments (after October 7, 2001) separately and duration of each deployment was calculated from deployment start and end dates. These were summed for a cumulative deployment length in days. Exposure to burn pit smoke (“Were you near a burn pit during these dates”) was queried for all deployments after October 7, 2001. Participants reported average number of hours of burn pit smoke exposure per day for each deployment segment which was multiplied by the number of days for that deployment. These values were summed for cumulative hours of burn pit smoke exposure over all deployments (“Cumulative Burn Pit Smoke”) which was divided by 24 h/day and average month length (365.25/12) to produce cumulative months of burn pit smoke exposure.

Other self-reported military exposures and activities included: heavy smoke from weapons, signal smoke, markers, or other combat items (“Heavy Smoke”); work in convoys or other vehicle operations (“Convoy”); perform refueling operations (“Refueling”); perform aircraft, generator, or other large engine maintenance (“Engine Maintenance”); perform construction duties (“Construction”); perform pesticide duties (“Pesticides”); and dust storms (“Dust Storms”). Participants reported these exposures as a duration of 0–31 days per month on average over all deployments. Cumulative total exposure was calculated from the average number of days per month (0–31 days) multiplied by the total number of months deployed (cumulative number of days deployed divided by 31 days/month). The group ’Unknown’ includes the responses of ‘I do not know’ and ‘Do not wish to answer’, as well as missing responses. In recognition that there could be differences between cases and controls with regard to the time since last deployment that might impact the effective ascertainment period, we compared time since last deployment to the end of the ascertainment period (“Time since last deployment”).

#### Covariates

Age in years, a continuous variable, was calculated from date of birth relative to questionnaire completion date from DoD personnel service records migrated into the AHOBPR. Data on sex (male/female/missing), race (White, Black, Asian, Native American, Native Hawaiian, Unknown) and ethnicity (Hispanic, Non-Hispanic, Unknown) was obtained from DoD records and supplemented with VHA data, if missing. Branch of service (Army, Navy, Air Force, Marine Corps) was determined based on the branch during the participant’s last deployment from DoD personnel records. Body mass index (BMI, kg/m^2^) was calculated from self-reported height and weight on the AHOBPR questionnaire. BMI was treated as a continuous variable in the regression model; missing and non-physiologic values were labelled “Other”.

Smoking of cigarettes was categorized as ‘never, past, current, and unknown,’ constructed from several AHOBPR items. Participants who self-reported smoking less than 100 cigarettes in their lifetime were considered never smokers. Past smokers reported smoking 100 or more cigarettes in their lifetime but did not endorse current smoking. Endorsement of smoking every day or some days was classified as a current smoker. Participants who stated, ‘I do not know’ and ‘do not wish to answer’ to smoking status, as well as those with no response, were classified as ‘Unknown.’

### Statistical analysis

A descriptive statistical analysis was performed for all variables by stratifying cases and controls. Bivariate analyses, *x*^2^ for categorical variables and *t*-test for continuous variables, were performed to assess differences. Multivariable logistic regression of matched cases and controls, unadjusted and adjusted for pertinent demographic factors (race, ethnicity, military service branch, BMI, and smoking status) tested for associations between sarcoidosis case status (yes vs. no) and exposure variables of interest (described above). We report odds ratios (ORs) and 95% confidence intervals (95% CI’s) for each variable included in the model. ORs for cumulative deployment-related exposures were reported in 6 month increments to ease interpretation and better match the experience of typical deployment durations.

## Results

From 212,427 individuals who were deployed to Southwest Asia, Afghanistan or Djibouti after October 7, 2001, we identified 177,718 AHOBPR participants who completed the AHOBPR by August 15, 2020, and had one or more encounters documented in VHA records. From that denominator we identified 661 veterans (0.37%) with an ICD code for sarcoidosis between 2011–2020. They were matched by sex and age to 2641 control AHOBPR participants who used VHA during the same period and had no ICD codes for sarcoidosis.

Bivariate analyses results are reported in Table 1. Participants with sarcoidosis were more frequently Black (42% vs. 12% of controls), non-Hispanic (94% vs. 61% of controls), former smokers (26% vs. 17% of controls), and Army veterans (70% vs. 64% of controls). Notably, participants with sarcoidosis were less frequently Navy veterans (6.7% vs. 12% of controls). The mean BMI was higher among those with sarcoidosis (30.3 kg/m^2^ vs. 29.1 kg/m^2^ in controls). The time since last deployment demonstrated a difference between cases (mean 14.7 (standard deviation 7.3) years) and controls (mean 12.2 (SD 6.4) years; p < 0.001).

**Table 1:** Characteristics of airborne hazards and open burn pit registry participants with sarcoidosis documented by international classification of disease (ICD) code in Veterans Health Administration electronic medical record data (n = 661) and age- and sex-matched controls (n = 2641).

Characteristic	Sarcoidosis diagnosis		Bivariate logistic regression		
	No N = 2641	Yes N = 661	OR	95% CI	p-value
	Mean (SD)	Mean (SD)			
**Age (years)**	49.0 (8.5)	49.1 (8.5)	1.00	0.99, 1.01	> 0.9
**Time since deployment (years)**	12.2 (6.4)	14.7 (7.3)	1.05	1.04, 1.07	< 0.001
**Body mass index (kg/m**^**2**^**)**	29.1 (4.6)	30.3 (5.2)	1.05	1.03, 1.07	< 0.001
Other	103 (3.9%)	34 (5.1%)			

	N (%)	N (%)			
**Gender**					
Male	2302 (87%)	576 (87%)	–	–	
Female	339 (13%)	85 (13%)	1.00	0.77, 1.29	> 0.9
**Race**					
White	1441 (55%)	356 (54%)	–	–	
Black	315 (12%)	275 (42%)	3.53	2.90, 4.31	< 0.001
Asian	310 (12%)	7 (1.1%)	0.09	0.04, 0.18	< 0.001
Native American	24 (0.9%)	5 (0.8%)	0.84	0.28, 2.05	0.7
Native Hawaiian	118 (4.5%)	5 (0.8%)	0.17	0.06, 0.38	< 0.001
Unknown	433 (16%)	13 (2.0%)	0.12	0.07, 0.20	< 0.001
**Ethnicity**					
Non-Hispanic	1624 (61%)	621 (94%)	–	–	
Hispanic	734 (28%)	22 (3.3%)	0.08	0.05, 0.12	< 0.001
Unknown	283 (11%)	18 (2.7%)	0.17	0.10, 0.26	0.001
**Service branch**					
Army	1681 (64%)	466 (70%)	–	–	
Air force	413 (16%)	100 (15%)	0.87	0.68, 1.11	0.3
Marines	234 (8.9%)	51 (7.7%)	0.79	0.57, 1.07	0.14
Navy	313 (12%)	44 (6.7%)	0.51	0.36, 0.70	< 0.001
**Smoking status**					
Never	1641 (62%)	370 (56%)	–	–	
Former	439 (17%)	171 (26%)	1.73	1.40, 2.13	< 0.001
Current	249 (9.4%)	60 (9.1%)	1.07	0.78, 1.44	0.7
Unknown	312 (12%)	60 (9.1%)	0.85	0.63, 1.14	0.3

*SD* standard deviation, *OR* odds ratio, *CI* confidence interval; “Other” for Body Mass Index represents missing and non-physiologic values.

Bivariate analysis of cumulative exposures by sarcoidosis status indicated no statistically significant differences in mean duration of any exposure (Table 2). Mean duration of exposure was generally lower among those with sarcoidosis than controls except for cumulative duration of convoy activities (cases 7.8 (SD 9.2) months vs. controls 7.7 (SD 9.4) months).

**Table 2:** Bivariate association between sarcoidosis and cumulative deployment-related exposures (mean (SD) of months over all deployments after October 2001) for cases (n = 661) and age- and sex-matched controls (n = 2641) from the Airborne Hazards and Open Burn Pit Registry participants who used the Veterans Health Administration.

Characteristic	Sarcoidosis diagnosis		Bivariate logistic regression		
	No N = 2641	Yes N = 661	OR	95% CI	p-value
	Mean (SD) N (%)	Mean (SD) N (%)			
**Burn pit smoke**	6.7 (7.1)	6.3 (7.2)	0.96	0.88, 1.04	0.3
Unknown	519 (19.7%)	156 (23.6%)			
**Heavy smoke**	8.4 (10.2)	7.5 (9.4)	0.94	0.88, 1.00	0.061
Unknown	580 (22.0%)	163 (24.7%)			
**Convoy**	7.7 (9.4)	7.8 (9.2)	1.01	0.95, 1.06	0.8
Unknown	226 (8.6%)	57 (8.5%)			
**Refueling**	6.5 (9.4)	6.4 (9.4)	0.99	0.93, 1.05	0.7
Unknown	267 (10.1%)	69 (10.4%)			
**Engine maintenance**	4.9 (9.1)	4.5 (8.7)	0.97	0.91, 1.03	0.3
Unknown	241 (9.1%)	60 ((9.1%)			
**Construction**	2.4 (5.7)	2.2 (5.4)	0.95	0.85, 1.05	0.4
Unknown	322 (12.2%)	86 (13.0%)			
**Pesticide**	0.8 (3.4)	0.7 (3.2)	0.93	0.76, 1.10	0.4
Unknown	396 (15.0%)	89 (13.4%)			
**Dust storm**	5.0 (6.7)	4.8 (6.7)	0.98	0.90, 1.06	0.7
Unknown	376 (14.2%)	101 (15.2%)			

SD = Standard Deviation, OR = Odds Ratio, CI = Confidence Interval, n (%) is reported for “unknown” responses for each exposure.

Fully adjusted logistic regression analysis (Table 3) demonstrated lower odds of sarcoidosis in participants who were Hispanic compared to non-Hispanics (OR 0.08, CI 0.04–0.15), and served in the Navy compared with participants in the Army (OR 0.43, CI 0.23–0.78). In contrast, veterans who were Black had higher odds of sarcoidosis when compared to veterans who were White (OR 2.28, CI 1.66–3.13). Former smokers were at higher risk of sarcoidosis compared to non-smokers (OR 1.89, CI 1.34–2.66).

**Table 3:** Multivariable logistic regression model reporting odds of association between sarcoidosis and cumulative deployment related exposures (in 6-month increments) and participant characteristics among 661 cases and 2641 age- and sex-matched participants from the Airborne Hazards and Open Burn Pit Registry cohort who used the Veterans Health Administration.

Characteristic	OR	95% CI	p-value
**Cumulative burn pit smoke**	0.94	0.80, 1.09	0.4
**Cumulative heavy smoke**	0.90	0.78, 1.05	0.2
**Cumulative convoy**	1.16	1.00, 1.35	0.046
**Cumulative refueling**	0.98	0.84, 1.15	0.8
**Cumulative engine maintenance**	1.04	0.91, 1.18	0.6
**Cumulative construction**	1.10	0.92, 1.31	0.3
**Cumulative pesticide**	0.98	0.74, 1.29	0.9
**Cumulative dust storm**	0.98	0.82, 1.17	0.8
**Race**			
White	–	–	
Black	2.28	1.66, 3.13	< 0.001
Asian	0.09	0.02, 0.21	< 0.001
Native American	0.82	0.18, 2.77	0.8
Native Hawaiian	0.11	0.02, 0.36	0.003
Unknown	0.22	0.09, 0.48	< 0.001
**Ethnicity**			
Non-Hispanic	–	–	
Hispanic	0.08	0.04, 0.15	< 0.001
Unknown	0.46	0.20, 0.98	0.052
**Service branch**			
Army	–	–	
Air force	1.16	0.77, 1.73	0.5
Marines	0.76	0.44, 1.27	0.3
Navy	0.43	0.23, 0.78	0.008
**Body mass index (kg/m**^2^**)**	1.02	0.99, 1.05	0.2
**Smoking status**			
Never	–	–	
Former	1.89	1.34, 2.66	< 0.001
Current	0.97	0.60, 1.52	0.9
Unknown	1.06	0.62, 1.77	0.8
**Time since deployment (years)**	1.03	1.01, 1.05	0.004

*OR* odds ratio, *CI* confidence interval.

Only cumulative convoy exposure was associated with sarcoidosis diagnosis in the multivariable logistic regression model (Table 3). Participants who performed convoy activities also had the highest odds of being cases compared to controls (OR 1.16, CI 1.00–1.35, p-value 0.046). The exposure variables were scaled such that odds ratios reflect difference in risk in 6-month increments. The point estimates for the association between sarcoidosis and all other exposures were smaller and non-significant (ORs 0.90–1.10, p-values 0.2–0.9).

We post hoc explored the possibility of statistically significant interactions between cumulative burn pit smoke exposure and two variables associated with sarcoidosis: race and smoking status. Neither interaction was statistically significant (see supplemental materials).

## Discussion

Among veterans who served in Iraq, Afghanistan, and/or Djibouti after 2001, who utilized VHA one or more times between 2011–2020, and completed the AHOBPR between 2014–2020, we identified 661 with a diagnosis of sarcoidosis documented in the VHA medical record. In a multivariable model of self-reported military exposures, race, ethnicity, military branch, body mass index, time since last deployment, and smoking status, we found that African American race, non-Hispanic ethnicity, and former smoking status were associated with increased risk of sarcoidosis diagnosis while those who reported military service in the Navy were less likely to have sarcoidosis (compared to Army service). We found only self-reported cumulative convoy activity exposure independently associated with sarcoidosis in the adjusted model.

Participants of African American race, non-Hispanic ethnicity, and higher body mass index were associated with greater odds of a sarcoidosis diagnosis. These characteristics have been consistently identified as risk factors for incident sarcoidosis in epidemiologic studies^20,21^. The relationship between smoking and sarcoidosis is more complex due to their temporal dependence—individuals suffering from symptoms due to undiagnosed condition or those newly diagnosed with a chronic respiratory disease may be more likely to stop smoking, for example. However, most studies have identified a higher risk of sarcoidosis in never or former smokers, which is partially reflected in our data^22,23^. Concordant identification of these risk factors in this investigation with regard to published literature supports the validity of the study design, and it suggests that null associations with other factors may reflect their true relationship in the study population, AHOBPR respondents who utilize the VHA. Extrapolation of these findings to the broader population of deployed veterans is not appropriate due to the voluntary nature of participation in the AHOBPR.

The AHOBPR questionnaire explicitly asks respondents to report exposure to a list of factors which may be plausibly associated with lung health: burn pit smoke; heavy artillery and machinery smoke; convoy activity and vehicle operations; refueling; engine maintenance; construction; pesticide application duties; and dust storms. Only the association between sarcoidosis and convoy activity during deployment was statistically significant (OR 1.16, CI 1.00–1.35, p-value 0.046), suggesting hypotheses that could be pursued in future research. The actual wording of the survey item was “In a typical month, how many days were you in convoy or other vehicle operations?” a fairly non-specific question suggesting the need for more detailed approaches to exposure assessment relevant to this domain, e.g., location in vehicle, specific role/activities, availability/use of vehicle climate control, external environmental conditions. While it is possible that a larger sample might detect a small association between sarcoidosis and other examined exposures, in general, case–control studies overestimate associations, suggesting that the observed lack of associations may be robust to larger samples.

Supporting this conclusion, Forbes and colleagues examined sarcoidosis incidence rates among active-duty Army personnel from 2005 to 2010 and reported no difference between those deployed to Southwest Asia compared to those who were never deployed, suggesting that in-theater exposures may not meaningfully increase the risk of developing sarcoidosis^24^. Of note, the association between Navy service and lower odds of sarcoidosis is consistent with a recent study of Military Health System data showing that the Navy accounted for the lowest proportion of sarcoidosis cases compared to the other service branches over 2004–2013^25^. Thus, while sailors have historically been considered at elevated risk for sarcoidosis, analysis of more recent data suggest the observed elevation of risk among Naval personnel may have peaked during the Vietnam era (1960s–1970s)^16,26–28^. Analysis of larger groups of military personnel across branches will be necessary to better answer these questions and to elucidate exposure- or task-specific risks of sarcoidosis.

This report is the first published analysis of AHOBPR data linked to VHA medical record data to explore risk factors for a disease not self-reported on the AHOBPR questionnaire. Leveraging the AHOBPR to characterize potential associations between burn pit and military-related environmental exposures and adverse health outcomes can inform operational harm reduction efforts and the design and implementation of post-deployment surveillance and treatment programs. Earlier analyses of AHOBPR have demonstrated its potential for detecting associations within the population of participants^26^ and the consistency of findings comparing self-reported, and clinician documented diagnoses^27^. However, registry-only approaches are limited by reliance on self-report and selection biases from voluntary participation which is consistent with recommendations from the National Academies of Sciences, Engineering and Medicine (NASEM)^28^. Our analysis demonstrates this approach using a case–control analysis nested within the cohort of AHOBPR participants. A strength of this study and the use of the AHOBPR data is the relatively large cohort of individuals with sarcoidosis (n = 661) with available medical record data and self-reported exposure information and well-matched controls.

There are several limitations of this report as enumerated in the NASEM review of the AHOBPR^28^. First, the AHOBPR is a voluntary activity and captures only a fraction of all potentially eligible veterans. Our cases were identified from the cohort participants as of August 15, 2020, representing only 212,427 participants out of an estimated 3.5 million eligible (see the NASEM report for information about the entire AHOBPR cohort)^28^. As such, there are likely several biases inherent in the data; eligible service members and veterans with greater concern about their exposures, those who are less healthy, and those with fewer alternatives to care may be more likely to participate in the AHOBPR. Second, while participants are presented with deployment segments from DoD records, the information gathered from the AHOBPR is self-reported, including information about any missed deployment segments. For these reasons, we do not fully discount the possibility of a clinically significant relationship between certain types of military exposures and sarcoidosis. Because of the self-reported and retrospective nature of the AHOBPR questionnaire, it is plausible that substantial inaccuracies in classification of the intensity and types of exposure may have masked a true association. Future analyses to address this limitation must incorporate data sources beyond the AHOBPR, including objective exposure ascertainment. On the other hand, because sarcoidosis was not a listed condition, we relied on documentation in the VHA medical record to classify cases and controls; a relative strength of this analysis. This does raise a third limitation of the study, however; we only included AHOBPR participants who had utilized the VHA one or more times to ensure accurate disease classification. Therefore, our findings cannot be applied to veterans who do not use the VHA. It is also well-established that disease codes captured at the time of care are not always accurate, another limitation of our approach. Finally, race and ethnicity is unknown for some cases and controls, although the proportions are relatively low for VHA and DoD data^29^.

Although it is unlikely that the diagnosis of sarcoidosis occurred prior to deployment, we were unable to confirm the exact onset or initial diagnosis outside the VHA medical record due to the retrospective nature of our analysis. To address potential differential time for recognition of sarcoidosis, we examined the time from last deployment to the end of the case status ascertainment period. Due to the difference in mean time between cases (14.7 (SD 7.3) years) and controls (12.2 (SD 6.4) years) (p < 0.001), we included this variable as a covariate in the final model. We were unable to address the possibility of differential reliance on VHA services between cases and controls which might have increased or decreased the likelihood of documentation of a sarcoidosis diagnosis.

Our findings of an association between race and ethnicity and sarcoidosis are consistent with published literature, suggestive of external validity. This corroboration of a well-established risk factor of sarcoidosis supports our conclusion that, in this cohort of AHOBPR participants who use the VHA, the examined military exposures are not prominently associated with sarcoidosis. These results suggest the need for more targeted explorations of specific exposures of concern using rigorous epidemiologic approaches and objective assessments of exposure. Advancing our understanding of the genetic contributions and gene by environment interactions relevant to sarcoidosis, including military exposures, may also be a fruitful direction for future research. The AHOBPR and linkable data available from the VHA and Department of Defense may provide unique opportunities for large scale investigations to test hypothesized associations among genetic, exposure and other individual factors and sarcoidosis.

## Supplementary Information

Supplementary Table 1.

## Data Availability

Data were obtained from the VA/DoD Airborne Hazards and Open Burn Pit Registry stewarded by the U.S. Department of Veterans Affairs Airborne Hazards and Burn Pits Center of Excellence. Requests for data should be directed to this entity.
