# Supplementary material for: Factors associated with a diagnosis of sarcoidosis among US veterans of Iraq and Afghanistan
Source: Sci Rep. 2022 Dec 21;12:22045. doi: 10.1038/s41598-022-24853-8 (PMC9772322; doi:10.1038/s41598-022-24853-8)
Supplement: Supplementary file 1 — Supplementary Table 1. [file 41598_2022_24853_MOESM1_ESM.docx]

Supplemental Table. Multivariable logistic regression models reporting association between sarcoidosis and cumulative deployment related exposures (in 6-month increments) and patient characteristics among age- and sex-matched participants from the Airborne Hazards and Open Burn Pit Registry cohort who used the Veterans Health Administration. The “Burn Pit Smoke X Race” model includes interaction terms for the cumulative burn pit smoke variable and race variable categories. The “Burn Pit Smoke X Smoking” model includes interaction terms for the cumulative burn pit smoke variable and smoking status variable categories.

|  | **Burn Pit Smoke X Race** | | | **Burn Pit Smoke X Smoking** | | |
| --- | --- | --- | --- | --- | --- | --- |
| Characteristic | OR | 95% CI | p-value | OR | 95% CI | p-value |
| **Cumulative Heavy Smoke** | 0.90 | 0.78, 1.04 | 0.2 | 0.92 | 0.79, 1.06 | 0.2 |
| **Cumulative Convoy** | 1.16 | 1.00, 1.35 | 0.049 | 1.14 | 0.98, 1.33 | 0.086 |
| **Cumulative Refueling** | 0.99 | 0.85, 1.15 | 0.9 | 0.97 | 0.83, 1.14 | 0.7 |
| **Cumulative Engine Maintenance** | 1.04 | 0.91, 1.18 | 0.6 | 1.05 | 0.92, 1.20 | 0.5 |
| **Cumulative Construction** | 1.10 | 0.92, 1.32 | 0.3 | 1.10 | 0.92, 1.31 | 0.3 |
| **Cumulative Pesticide** | 0.97 | 0.73, 1.29 | 0.8 | 0.99 | 0.74, 1.31 | >0.9 |
| **Cumulative Dust Storm** | 0.98 | 0.82, 1.17 | 0.8 | 1.00 | 0.83, 1.19 | >0.9 |
| **Ethnicity** |  |  |  |  |  |  |
| Non-Hispanic | — | — |  | — | — |  |
| Hispanic | 0.08 | 0.04, 0.15 | <0.001 | 0.08 | 0.04, 0.15 | <0.001 |
| Unknown | 0.46 | 0.20, 0.98 | 0.053 | 0.45 | 0.19, 0.95 | 0.045 |
| **Service Branch** |  |  |  |  |  |  |
| Army | — | — |  | — | — |  |
| Air Force | 1.16 | 0.77, 1.74 | 0.5 | 1.13 | 0.75, 1.69 | 0.6 |
| Marines | 0.76 | 0.44, 1.27 | 0.3 | 0.72 | 0.42, 1.22 | 0.2 |
| Navy | 0.43 | 0.23, 0.78 | 0.008 | 0.43 | 0.23, 0.78 | 0.008 |
| **Body Mass Index (kg/m^2^)** | 1.02 | 0.99, 1.05 | 0.2 | 1.02 | 0.99, 1.05 | 0.2 |
| **Time Since Deployment (years)** | 1.03 | 1.01, 1.05 | 0.004 | 1.03 | 1.01, 1.05 | 0.005 |
| **Smoking Status** |  |  |  |  |  |  |
| Never | — | — |  | — | — |  |
| Former | 1.89 | 1.34, 2.66 | <0.001 | 1.48 | 0.93, 2.34 | 0.092 |
| Current | 0.96 | 0.60, 1.51 | 0.9 | 0.79 | 0.42, 1.43 | 0.4 |
| Unknown | 1.07 | 0.62, 1.78 | 0.8 | 1.74 | 0.78, 3.89 | 0.2 |
| **Cumulative Burn Pit Smoke** | 0.92 | 0.76, 1.09 | 0.4 | 0.87 | 0.71, 1.06 | 0.2 |
| **Race** |  |  |  |  |  |  |
| White | — | — |  | — | — |  |
| Black | 2.15 | 1.40, 3.30 | <0.001 | 2.24 | 1.63, 3.08 | <0.001 |
| Asian | 0.13 | 0.03, 0.45 | 0.004 | 0.08 | 0.02, 0.21 | <0.001 |
| Native American | 0.87 | 0.11, 5.70 | 0.9 | 0.81 | 0.17, 2.81 | 0.8 |
| Native Hawaiian | 0.05 | 0.00, 0.37 | 0.014 | 0.10 | 0.02, 0.34 | 0.002 |
| Unknown | 0.20 | 0.06, 0.57 | 0.004 | 0.22 | 0.09, 0.48 | <0.001 |
| **Cumulative Burn Pit Smoke * Race** |  |  |  |  |  |  |
| Burn Pit Smoke * Black | 1.06 | 0.81, 1.39 | 0.7 |  |  |  |
| Burn Pit Smoke * Asian | 0.64 | 0.11, 1.75 | 0.5 |  |  |  |
| Burn Pit Smoke * Native American | 0.94 | 0.10, 3.15 | >0.9 |  |  |  |
| Burn Pit Smoke * Native Hawaiian | 1.83 | 0.40, 6.51 | 0.3 |  |  |  |
| Burn Pit Smoke * Unknown | 1.11 | 0.46, 2.05 | 0.8 |  |  |  |
| **Cumulative Burn Pit Smoke * Smoking Status** |  |  |  |  |  |  |
| Burn Pit Smoke * Former |  |  |  | 1.27 | 0.94, 1.72 | 0.12 |
| Burn Pit Smoke * Current |  |  |  | 1.22 | 0.84, 1.75 | 0.3 |
| Burn Pit Smoke * Unknown |  |  |  | 0.59 | 0.26, 1.11 | 0.2 |
| OR = Odds Ratio, CI = Confidence Interval | | | | | | |
